# Prevalence of Parasitic Disease Burden in the Adult Population Presenting With Persistent or Chronic Diarrhea

**DOI:** 10.7759/cureus.71959

**Published:** 2024-10-20

**Authors:** Mehwash Iftikhar, Mian Mufarih Shah, Sheraz J Khan, Imran Khan, Muhammad Bilal Khattak, Nazir Shah, Saeed Ur Rahman

**Affiliations:** 1 Medicine, Medical Teaching Institution-Hayatabad Medical Complex Peshawar, Peshawar, PAK; 2 Internal Medicine, Medical Teaching Institution-Hayatabad Medical Complex Peshawar, Peshawar, PAK; 3 Medicine, Khyber Girls Medical College, Peshawar, PAK; 4 Pathology, Medical Teaching Institution-Hayatabad Medical Complex Peshawar, Peshawar, PAK

**Keywords:** adult population, chronic diarrhea, intestinal parasitic infection, persistent diarrhea, prevalence

## Abstract

Introduction

Parasitic infection is an overlooked cause of diarrhea in adults. It can cause persistent or chronic diarrhea that contributes to a significant burden on the overall morbidity of the population. Stool sampling would aid in the diagnosis of parasitic infection in adults presenting with diarrhea.

Methods

A cross-sectional study was conducted from January to June 2024 at Medical Teaching Institution (MTI)-Hayatabad Medical Complex, Peshawar, Pakistan. A total of 500 stool samples were collected using non-probability consecutive sampling. All the patients presenting with complaints of persistent or chronic diarrhea to the medical outpatient department or admitted to the medical units were included in the study. Patients with bloody diarrhea and those less than 13 years of age were excluded from the study. Verbal and written informed consent was obtained from all the patients included in the study. The stool samples of all the patients were collected, reported, and verified by the microbiology department.

Results

Out of 500 stool samples of the patients with persistent or chronic diarrhea, 174 (34.8%) were found to be infected with cysts or trophozoites of parasites. Gender distribution of parasitic infections showed that 89 out of 245 females (36.3%) and 85 out of 255 males (33.3%) were affected. The comparison between genders yielded a p-value of 0.482. All the patients showed mono parasitism. The most common isolate was *Giardia lamblia* in 90 (51.72%) cases, followed by H-Nana in 49 (28.17%) isolates, *Entamoeba histolytica* in 16 (9.20%), *Ascaris lumbricoides* in 14 (8.04%), *Trichuris trichura* in two (1.15%), *Taenia saginata *in two (1.15%), and *Cryptosporidium* in one (0.6%) infected patient.

Conclusion

Parasitic intestinal infections, particularly *Giardia* and H-Nana, are prevalent yet overlooked causes of persistent and chronic diarrhea in adults. These findings underscore the importance of routine stool examination as a cost-effective diagnostic tool, potentially improving patient outcomes and reducing unnecessary medical interventions.​​​​​​​​​

## Introduction

Diarrhea ranks eighth among the top 10 global causes of death, as reported by the Global Health Estimates 2020, by the World Health Organization (WHO). It is classified based on symptom duration as acute (<2 weeks), persistent (two to four weeks), and chronic (>4 weeks) [1]. Chronic diarrhea affects over 5% of the population per year, with 40% of cases occurring in individuals above 60 years of age [2,3]. Persistent diarrhea affects approximately 3% of travelers to developing countries [1-6].

Intestinal parasitic infections are considered a neglected tropical disease [5]. These infections are a significant cause of morbidity, particularly in children, and are more prevalent in developing countries [7]. In adults, parasitic infections have been primarily studied in relation to traveler’s diarrhea, often associated with tropical and sub-tropical regions [8,9]. Despite being traditionally linked to impoverished areas, parasitic infections have garnered attention in developed countries due to increased international travel, human-animal migration, and the rising number of immunocompromised individuals and elderly populations [1]. This concern is particularly notable among diabetic patients, who form a key subset of the immunocompromised population and are susceptible to parasitic-related diarrhea [10].

*Giardia lamblia* (also known as *G. duodenalis* or *G. intestinalis*) is the most common etiological agent of chronic diarrhea worldwide [11], with prevalence rates ranging from 2.75% to 9.5% in Pakistan [12]. While people of all ages are affected by parasitic infections, children remain the most vulnerable group. Recent studies have also suggested a rise in infection rates of *G. lamblia *and *Blastomyces hominis* in the post-pandemic period [9]. This study aims to determine the prevalence and burden of parasitic infections in the adult population presenting with persistent or chronic diarrhea.

## Materials and methods

Study design and setting

This study was a cross-sectional, prospective analysis conducted from January 1 to June 30, 2024, at the Medical B Unit of Medical Teaching Institution (MTI)-Hayatabad Medical Complex, Peshawar, Pakistan. The study included both outpatients and inpatients from the surrounding region who presented with persistent or chronic diarrhea.

Study population

A total of 500 participants were selected based on a prevalence estimation using the formula n = Z² * P(1-P) / d², where Z = 1.96 for a 95% confidence level, P = 0.30 (estimated prevalence of 30% based on previous studies), and d = 0.04 (margin of error). This calculation yielded a sample size of approximately 504, which we rounded to 500 for practical purposes. 

 Inclusion Criteria

The study included male and female patients aged 13 years or older who presented with persistent or chronic diarrhea, which was defined as diarrhea lasting between two and four weeks, and chronic diarrhea as diarrhea lasting more than four weeks, in accordance with the WHO guidelines. All patients who provided informed consent for participation in the study were eligible for inclusion.

Exclusion Criteria

Patients were excluded from the study if they were currently receiving antibiotic, antiparasitic, or immunosuppressive therapy, including HIV-positive patients. In addition, those with underlying gastrointestinal diseases such as inflammatory bowel disease or irritable bowel syndrome were not included. Patients who were unable or unwilling to provide stool samples were also excluded from participation.​​​​​​​​​

Sampling technique

A non-probability convenience sampling technique was used to select patients visiting the outpatient department or admitted to the ward during the study period. All eligible patients meeting the inclusion criteria were invited to participate.

Data collection and sample analysis

After obtaining informed consent, stool samples were collected from all enrolled patients. The stool specimens were examined using standard laboratory protocols, including both gross examination and microscopic analysis for parasitic organisms. Microscopy was performed to detect parasites based on their morphology. All examinations were conducted by trained laboratory personnel following standard operating procedures.

Statistical analysis

Data were entered and analyzed using IBM SPSS Statistics for Windows, Version 23.0 (IBM Corp., Armonk, NY). Descriptive statistics were calculated, including frequencies and percentages for categorical variables. The chi-square test and independent sample T-test were used to assess associations between parasitic infection and demographic variables. A p-value of <0.05 was considered statistically significant.

Ethical considerations

Ethical approval for the study was obtained from the MTI-Hayatabad Medical Complex Hospital Research and Ethical Committee (IREB) (ref. 2019, dated 13-12-2023). Written informed consent was obtained from all participants prior to sample collection.

## Results

Table 1 presents the demographic characteristics of the study population, showing no statistically significant difference in parasitic infection between males and females (p = 0.482), with a balanced gender distribution. However, a significant association was found between educational status and parasitic infection (p = 0.000), as a higher proportion of illiterate individuals (90.8%) were infected compared to the educated group (9.2%). The mean age of the participants was 31.5 years (±2.64), and there was no significant difference in age between those with and without parasitic infection (p = 0.955), indicating that age does not appear to influence infection rates.

**Table 1:** Demographic characteristics of the study population *p < 0.05 is considered statistically significant.

Variable		Total (n = 500)	No parasite (n = 326)	Parasite (n = 174)	p-value
Gender	Female	245 (49.0)	156 (47.9)	89 (51.1)	0.482
	Male	255 (51.0)	170 (52.1)	85 (48.9)	
Educational status	Illiterate	321 (64.2)	163 (50.0)	158 (90.8)	0.000*
	Educated	179 (35.8)	163 (50.0)	16 (9.2)	
Age (Mean ± SD)		31.50 ± 2.64	31.53 ± 12.63	31.46 ± 12.69	0.955

Table 2 displays the distribution of intestinal parasites among the 174 participants who tested positive for parasitic infection. *G. lamblia* was the most prevalent parasite, affecting 51.72% of the infected population, followed by *Hymenolepis nana* at 28.17%. Other parasites included *Entamoeba histolytica *(9.20%), *Ascaris lumbricoides* (8.04%), *Trichuris trichura* (1.15%), *Taenia saginata *(1.15%), and *Cryptosporidium* (0.6%). The cumulative percentage of these infections increases sequentially, reaching 100% with the inclusion of *Cryptosporidium*.

**Table 2:** Intestinal parasites (n = 174)

Intestinal parasites	Frequency (n)	Percentage (%)	Cumulative percentage (%)
Giardia lamblia	90	51.72	51.72
Hymenolepis nana	49	28.17	79.88
Entamoeba histolytica	16	9.20	89.08
Ascaris lumbricoides	14	8.04	97.13
Trichuris trichura	2	1.15	98.27
Taenia saginata	2	1.15	99.43
Cryptosporidium	1	0.6	100

## Discussion

The prevalence and diversity of intestinal protozoan and helminth infections vary significantly across different regions and populations [8,13]. Various studies have reported varying prevalence rates of intestinal parasites in different countries, such as 28.7% in Saudi Arabia [13], 9.48% in Sudan [14], and 19% in Ethiopia [15]. The diversity of parasitic species also differed across these regions. In comparison to the widespread data on pediatric populations, research on adult populations is more limited, particularly in Pakistan and India. Our study adds to this gap by examining parasitic infections in adults with persistent or chronic diarrhea. The overall prevalence of intestinal parasitic infection in Pakistan is estimated to be between 20% and 40% [16-20]. Our study found a prevalence rate of 34.8%, which aligns with these estimates but is notably lower than the 45.3% reported in a study from Islamabad by Singh et al. and the 68.8% in a study conducted by Siddiqui et al. in Karachi. However, our results are higher than the 11.18% prevalence found in a recent study in Delhi [21].

The variation in prevalence between different studies could be due to several factors, including differences in study populations, environmental conditions, diagnostic methods, and healthcare access. This underscores the need for more standardized surveillance and reporting methods to facilitate comparisons across regions and populations. Moreover, our study found that 52.9% of those with parasitic infections (92 out of 174) had never undergone stool testing before, highlighting a critical gap in the diagnostic process for chronic diarrhea. This finding emphasizes the importance of routine stool testing, especially in regions where parasitic infections are prevalent but often overlooked.

Our study also found that 22 patients with diabetic gastropathy and diarrhea were diagnosed with intestinal parasitic infections. In these cases, symptoms were initially attributed solely to diabetic gastropathy, leading to a delay in the diagnosis of parasitic infection. This is consistent with findings from Wala Ramadan et al., who reported a higher prevalence of parasitic infections in diabetic patients with diarrhea in Egypt. While our study does not conclusively establish a direct association between diabetic gastropathy and parasitic infection, it does suggest that parasitic infections may be underdiagnosed in this population, particularly among those presenting with chronic diarrhea.

This raises important implications for healthcare providers in regions with high burdens of intestinal parasitic infections. There is a clear need for increased awareness of the potential for parasitic infections to complicate other conditions, such as diabetes. Screening for parasitic infections should be integrated into the diagnostic process for chronic gastrointestinal symptoms, especially in immunocompromised populations. Our findings suggest that missed diagnoses could lead to unnecessary delays in treatment and prolonged morbidity.

In terms of prevention, more robust public health strategies are needed. Improved sanitation, access to clean water, and hygiene education remain critical in reducing the transmission of intestinal parasites. Regular screening programs, particularly for high-risk populations such as those with chronic gastrointestinal symptoms or immunocompromised states, should be implemented. In addition, healthcare providers should be trained to recognize and consider parasitic infections as a differential diagnosis, even in cases where symptoms may initially suggest other conditions, such as diabetic gastropathy.

Though our study focused mainly on parasitic infections, we certainly acknowledge that parasites may coexist with other microorganisms in causing persistent or chronic diarrhea. The complex interplay between parasites, bacteria, and viruses in the gut microbiome can influence the severity and duration of diarrheal symptoms. Future studies should consider a more comprehensive approach, investigating the full spectrum of potential pathogens better to understand their individual and combined roles in chronic diarrhea.

A notable limitation of our study is the inability to adequately account for potential biases or confounding variables, such as prior treatments and co-existing infections, which may have influenced the observed results. In addition, the reliance on a single stool specimen may have reduced parasite detection accuracy, as intermittent shedding can occur. Exclusion of dysentery cases and lack of screening for family members of infected patients limited our ability to assess broader transmission dynamics. Furthermore, low follow-up rates hindered the evaluation of treatment efficacy and symptom resolution. Recall bias, as well as previous anti-helminthic treatments, were not fully accounted for, potentially skewing results. Misdiagnoses were also a concern, particularly for diabetic patients who underwent expensive tests without stool examination. Finally, co-infections with bacteria or viruses were not explored, which could have contributed to the clinical symptoms. Future studies should address these gaps by using multiple stool samples, screening for co-infections, improving follow-up, and considering the growing issue of anti-helminth resistance in developing countries. Incorporating routine stool examinations into diagnostic protocols could improve patient outcomes and reduce unnecessary healthcare costs.

## Conclusions

This study highlights the significant role of parasitic infections, particularly *G. lamblia* and *H. nana*, in chronic diarrhea among adults, with a prevalence of 34.8%. It emphasizes the need for routine stool testing, an often-overlooked and cost-effective diagnostic tool, to improve diagnosis and treatment. The research calls for better awareness and training among healthcare professionals to prevent misdiagnoses, such as mistaking parasitic infections for conditions like diabetic gastropathy. Addressing resource limitations and enhancing diagnostic protocols are key to improving patient outcomes and reducing healthcare costs.
